# Spectral counting assessment of protein dynamic range in cerebrospinal fluid following depletion with plasma-designed immunoaffinity columns

**DOI:** 10.1186/1559-0275-8-6

**Published:** 2011-06-03

**Authors:** Jacques Borg, Alex Campos, Claudio Diema, Núria Omeñaca, Eliandre de Oliveira, Joan Guinovart, Marta Vilaseca

**Affiliations:** 1 Laboratoire de Neurobiochimie, Université Jean Monnet, Saint-Etienne, France; 2 Proteomics Platform, Barcelona Science Park, Barcelona, Spain; 3 Mass Spectrometry Core Facility, Institute for Research in Biomedicine, Barcelona, Spain; 4 Institute for Research in Biomedicine, Barcelona, Spain

**Keywords:** CSF, APEX, Biomarkers, depletion column, enrichment, low-abundance proteins

## Abstract

**Background:**

In cerebrospinal fluid (CSF), which is a rich source of biomarkers for neurological diseases, identification of biomarkers requires methods that allow reproducible detection of low abundance proteins. It is therefore crucial to decrease dynamic range and improve assessment of protein abundance.

**Results:**

We applied LC-MS/MS to compare the performance of two CSF enrichment techniques that immunodeplete either albumin alone (IgYHSA) or 14 high-abundance proteins (IgY14). In order to estimate dynamic range of proteins identified, we measured protein abundance with APEX spectral counting method.

Both immunodepletion methods improved the number of low-abundance proteins detected (3-fold for IgYHSA, 4-fold for IgY14). The 10 most abundant proteins following immunodepletion accounted for 41% (IgY14) and 46% (IgYHSA) of CSF protein content, whereas they accounted for 64% in non-depleted samples, thus demonstrating significant enrichment of low-abundance proteins. Defined proteomics experiment metrics showed overall good reproducibility of the two immunodepletion methods and MS analysis. Moreover, offline peptide fractionation in IgYHSA sample allowed a 4-fold increase of proteins identified (520 vs. 131 without fractionation), without hindering reproducibility.

**Conclusions:**

The novelty of this study was to show the advantages and drawbacks of these methods side-to-side. Taking into account the improved detection and potential loss of non-target proteins following extensive immunodepletion, it is concluded that both depletion methods combined with spectral counting may be of interest before further fractionation, when searching for CSF biomarkers. According to the reliable identification and quantitation obtained with APEX algorithm, it may be considered as a cheap and quick alternative to study sample proteomic content.

## Introduction

Biomarkers are key tools for detecting and monitoring neurodegenerative processes. Clinical Proteomics is especially well-suited to the discovery and implementation of biomarkers derived from biofluids. A major limiting factor for in-depth proteomics profiling is the immense dynamic range of biofluid proteins, which spans 10 to 12 orders of magnitude [1]. In human plasma, the 22 most abundant proteins are responsible for ~99% of the bulk mass of the total proteins, thus leaving several hundreds or thousands of proteins in the remaining 1%. Many biomarkers of "interest" are anticipated to be present at low concentrations and their detection is therefore hindered by highly abundant proteins. To overcome this problem, enrichment techniques and orthogonal fractionation strategies are routinely applied in proteomics studies prior to mass spectrometry (MS) analysis. Recent studies have demonstrated a substantial impact of multidimensional fractionation on the overall number of proteins identified and on sequence coverage [2-6]. Despite its benefits, extensive fractionation contributes to experimental variability and limits sample throughput.

Cerebrospinal fluid (CSF) in particular is directly related to the extracellular space of the brain and is therefore a valuable reporter of processes that occur in CNS. In the last few years, a number of proteomics strategies have been adopted to achieve in-depth coverage of the human CSF proteome. SCX-fractionation and LC-MALDI were used to identify 1,583 CSF proteins [2]. GeLC-MS/MS approach allowed identification of 798 proteins from albumin-depleted CSF [6]. Recently, combinatorial peptide ligand library was employed to decrease CSF dynamic range and identify 1,212 proteins [7]. In an attempt to generate a comprehensive CSF database, Pan et al. [8] combined and re-analyzed the results of various CSF proteomics studies and reported 2,594 unique proteins with high confidence.

A number of commercial depletion systems are available for highly selective removal of 1, 14, 20, or over 60 of the most abundant proteins present in human plasma. Although these systems were initially designed to deplete plasma/serum samples, they have been widely used for other biofluids such as CSF. A number of reports have evaluated the efficiency and reproducibility of these systems [9-15]. They have also pointed out the potential loss of non-target proteins as a result of non-specific binding to immunodepletion columns [10,12].

Here we evaluated the advantages afforded by immunodepletion and pre-fractionation of CSF samples. For this purpose, human CSF samples were analyzed after the removal of albumin or 14 HAP (high abundance protein) and were compared with non-depleted CSF samples without further offline fractionation. Noteworthy, the commercial depletion system used to remove 14 HAP was designed to stoichiometrically remove the 14 most abundant proteins in normal plasma/serum samples. Depleted samples were then analyzed by LC-MS/MS and further profiled using a modified spectral counting approach. In addition to proteome depth, we evaluated the performance of CSF enrichment and fractionation strategies in terms of reproducibility and experimental bias.

## Results

### Protein recovery after immunodepletion

**Figure 1:**
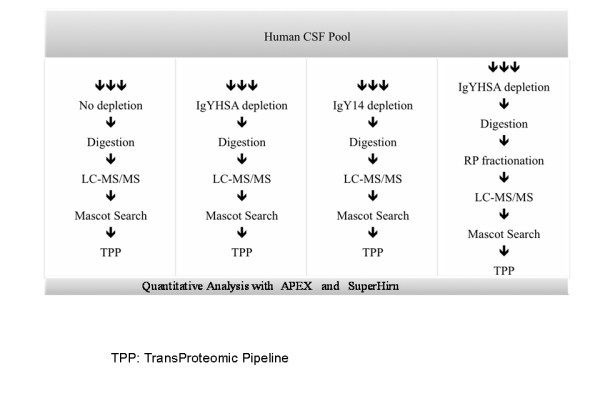
**Overview of the workflow used for CSF proteome analysis**. A pooled CSF sample was divided into 12 equal aliquots. Each aliquot was subjected to immunoaffinity protein depletion as follows: 14 proteins; albumin only; or were not subjected to depletion (controls). 75 μg of each flow-through (or non-depleted sample) was trypsin-digested and further analyzed by LC-MS/MS. MS raw data files were processed with Mascot Distiller and further analyzed with PeptideProphet algorithm. Protein abundance was calculated with APEX spectral counting method. Right-hand column shows analysis including reversed-phase LC peptide fractionation.

Figure 1 schematically illustrates the sample processing strategies adopted in this study. The amount of protein recovered in the flow-through (~ 3 or 4 mL for IgYHSA or IgY14 columns, respectively) following sample concentration with Amicon filters was around 13% and 30% of applied protein for the IgY14 and IgYHSA columns, respectively ( Table 1). Furthermore, the amount of protein recovered in the fractions bound to the IgY14 and IgYHSA columns was 52% and 37%, respectively.

**Table 1:** Total protein quantitation upon immunodepletion procedure.

	Before depletion (μg)	Flow-through fraction (μg)	Bound fraction (μg)
IgYHSA	780	248 ± 40	301 ± 25

IgY14	780	106 ± 2	425 ± 6

Protein quantification was carried out in triplicate in CSF samples depleted for 14 proteins (IgY14) or albumin (IgYHSA) with bicinchoninic acid colorimetric method. Results are shown as mean ± SD.

### Reproducibility

To evaluate the technical variability of immunodepletion strategies, a single pooled CSF sample was aliquoted and the assays were run as triplicates. Run-to-run reproducibility was evaluated using a set of proteomics experiment metrics. The number of MS1 and MS2 spectra acquired during the retention time period over which the middle 50% of the identified peptides elute, are direct measures of the effective speed of sampling during the most information-rich section of the run. Notably, the total number of MS1 and MS2 spectra was consistent across all samples (Table 2). The number of MS2 spectra was also reproducible between the three replicates of each method. Taken together, MS1 and MS2 scan counts metrics provide a broad perspective of the reliability of sample preparation and LC-MS performance for subsequent label-free quantitative analysis.

**Table 2:** Reproducibility of MS1 or MS2 spectral counts following various depletion methods.

	MS1 scans	MS2 scans
IgY14_1	787	3347

IgY14_2	933	3222

IgY14_3	911	3194

IgYHSA_1	783	2906

IgYHSA_2	778	2870

IgYHSA_3	606	2366

Undepleted_1	903	2372

Undepleted_2	1052	2781

Undepleted_3	1058	2888

Depleted or non-depleted CSF samples were analyzed as triplicates. Number of MS1 and MS2 scans over which the middle 50% of the identified peptides elute are shown for each CSF aliquot.

To evaluate pattern similarities across runs, we applied a label-free strategy based on matching features (m/z and retention time) across the three LC-MS replicates for each method. Briefly, features across replicate were mapped and aligned using SuperHirn algorithm, which clusters monoisotopic masses of the same charge state and m/z value (integration tolerance = 0.005 Da) across subsequent scans. Therefore, each feature is summarized by its m/z, retention time start/apex/end, and total feature area. Only features with charges 2+, 3+, 4+ and 5+ were considered in this analysis. In order to match two features between two or more replicates, we considered only features within 10 ppm and 60 s tolerance in m/z and retention time, respectively. Immunodepletion improved the final number of features found in the triplicate LC-MS analyses by approximately 20% (Table 3). Non-depleted samples presented slightly better reproducibility compared to the immunodepleted samples in terms of percentage of overlapping features among the three replicates (although lower in absolute number). Approximately 60% of all features detected in the non-depleted triplicates were found at least in 2 out of 3 replicates, whereas this number decreased to 55% in both immunodepletion techniques (Table 3). These observations demonstrate overall good reproducibility of the two immunodepletion methods.

**Table 3:** Pattern similarity following various depletion methods.

Method	Number of detected features	Number of common features in 3 replicates	Number of common features in 2 replicates	Number of features in only 1 replicate
IgY14	5478	1740 (31.8%)	1229 (22.4%)	2509 (45.8%)

IgYHSA	5446	1611 (29.5%)	1387 (25.5%)	2448 (45%)

Undepleted	4344	1465 (33.7%)	1124 (25.9%)	1755 (40.4%)

Table shows features (extracted and aligned with SuperHirn program) common to all 3 replicates in each depletion methods, those common to 2 replicates (excluding those common to the 3 replicates) and those found in only 1 replicate.

### Dynamic range

**Figure 2:**
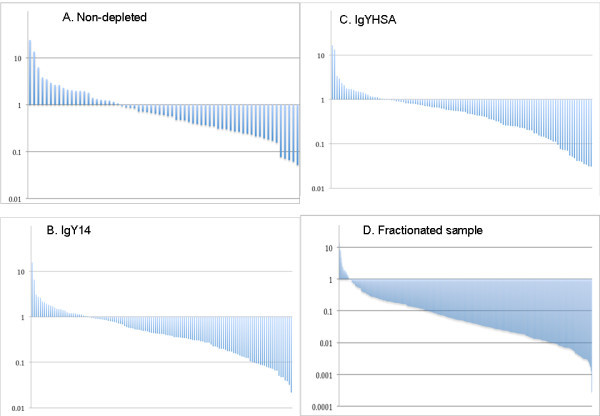
**Dynamic range of protein abundance**. Abundance of each identified protein was calculated with APEX algorithm. Abundance is plotted on log scale spanning 4 orders of magnitude. Proteins with an APEX value below 0.1log are considered LAP. Data shown were obtained from one typical set of data for each depletion method. A: non-depletion; B: IgY14-depletion; C: IgYHSA-depletion. D: IgYHSA-depletion and RP-fractionation.

Under the premise that spectral counting is correlated with peptide abundance [16,17], we evaluated the changes in CSF proteome content after depletion of highly abundant plasma proteins. Recently, the protein abundance calculated by APEX has been demonstrated to be a close approximation of the relative abundance of a particular protein [10]. Figure 2 shows a comparison of the dynamic range profile of CSF proteome achieved after immunodepletion as measured by APEX algorithm. Our data demonstrate an improvement in the overall number of low abundance proteins (LAP; below 2 logs of magnitude from the most abundant protein) in samples subjected to IgYHSA (14 proteins) or IgY14-depletion (18 proteins) compared to non-depleted (5 proteins) samples.

### Peptide and protein identification

As expected, the enrichment of LAP following immunodepletion significantly improved proteome coverage. The number of proteins identified increased after immunodepletion, particularly with IgY14 column (Table 4). A total of 665 unique peptides were confidently (PeptideProphet > 0.95) identified in the three IgYHSA replicates, of which 467 (70%) were found in at least two runs. Regarding IgY14 method, 775 unique peptides were confidently identified, of which 452 (58%) were identified in at least two replicates. Finally, for the non-depleted samples, a total of 466 peptides were confidently identified, of which 335 (72%) were common to at least two runs. Despite the improved proteome coverage achieved with the IgY14 depletion, there was a drop in the percentage of peptides identified in at least two replicates.

**Table 4:** Summary of peptide and protein identification after application of depletion methods and peptides prefractionation.

	Number of spectra identified	Number of unique peptides identified^1^	Number of proteins identified^2^
IgY14_1	893	571	136

IgY14_2	823	473	124

IgY14_3	881	463	120

**Total unique**		**775**	**156**

IgYHSA_1	837	518	105

IgYHSA_2	804	493	112

IgYHSA_3	652	366	84

**Total unique**		**665**	**131**

Undepl_1	724	277	67

Undepl_2	795	355	78

Undepl_3	773	384	75

**Total unique**		**466**	**90**

IgYHSA-RP30_1	15,992	2,470	433

IgYHSA-RP30_2	12,549	2,282	396

IgYHSA-RP30_3	12,381	2,164	390

**Total unique**		**3,026**	**535**

CSF samples were analyzed as triplicates following depletion of 14 proteins (IgY14), albumin only (IgYHSA) or no depletion (Undepl). Additionally CSF samples were analyzed after albumin depletion and further fractionation by reversed-phase liquid chromatography (IgYHSA-RP30).

1. only hits with Peptide Prophet ≥ 0.95

2. protein identification with Peptide Prophet ≥ 0.9.

**Figure 3:**
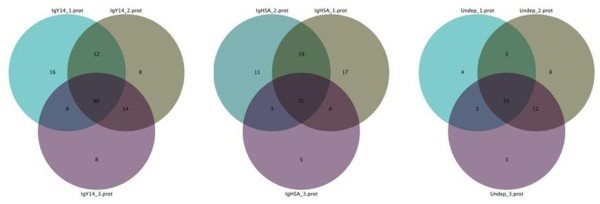
**Venn diagrams showing distribution of proteins identified in triplicate experiments after various depletion methods**.

At the protein level, we found 90 proteins common to the three IgY14 replicates from a total of 156 proteins; 72 proteins were common to all three IgYHSA replicates from a total of 131 proteins; and 55 proteins were common to all three non-depleted replicates from a total of 90 proteins (Figure 3). Overall, approximately 80% of the proteins identified in each method were found in at least 2 replicates.

**Figure 4:**
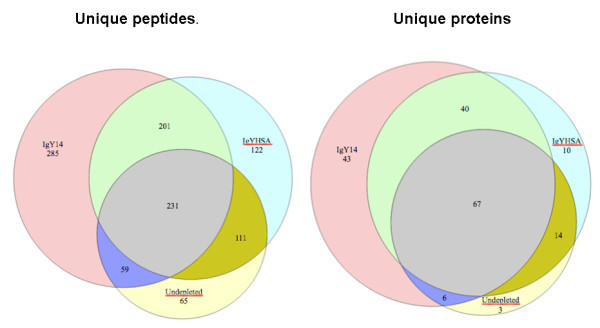
**Venn diagram showing distribution of unique peptides (left) and proteins (right) identified with various depletion methods with PeptideProphet confidence > 0.95**.

**Figure 5:**
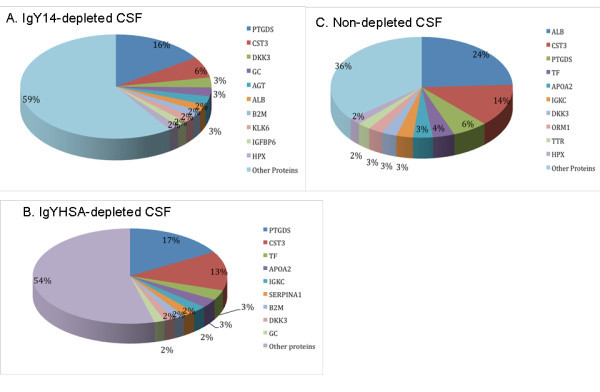
**Distribution of the 10 most abundant proteins identified in CSF in immunodepleted and non-depleted samples**. A: IgY14-depletion; B: IgYHSA-depletion. C: non-depletion. Protein abbreviations are as follows: AGT, Angiotensinogen; ALB, albumin; APOA2, Apolipoprotein A-II; B2 M, Beta-2-microglobulin; CST3, Cystatin-C; DKK3, Dickkopf-related protein-3; GC, Vitamin-D-binding protein; HPX, Hemopexin; IGFBP6, Insulin-like growth factor-binding protein-6; IGKC; KLK6, kallikrein-6; ORM1, orosomucoid-1; PTGDS, Prostaglandin-H2-D-isomerase; SERPINA1, Alpha-1-antitrypsin; TF, Serotransferrin; and TTR, Transthyretin.

Figure 4 shows the similarities in terms of peptide and protein identification across the three methods. 231 peptides and 67 proteins were commonly identified in the three methods, while 432 peptides and 107 proteins were commonly identified in both depleted samples. The differences between proteins identified in the IgYHSA-depleted replicates and undetected in the IgY14-depleted replicates are attributed, in part, to more proteins being targeted for depletion in the latter method. A manual inspection of the protein list not identified in samples subjected to IgY14 depletion indicates that 13 proteins (out of 24) were removed by IgY14 column (isoforms of haptoglobulin, fibrinogen, complement C3, and a number of immunoglobulin fragments). The lists of proteins and peptides identified are available as Additional File 1 Table S1 and Additional File 2 Table S2, respectively, along with corresponding protein abundance as calculated by APEX (Additional File 3 Tables S3, Additional File 4 Table S4 and Additional File 5 Table S5). The distribution of most abundant proteins showed that 9-10 proteins accounted each for more than 2% of total identified proteins (Figure 5). The 10 most abundant proteins following immunodepletion accounted for 41% (IgY14) and 46% (IgYHSA) of total CSF protein content, whereas they accounted for 64% of total protein content in non-depleted CSF samples. Except for abundant proteins common with plasma, our data also point out other proteins, such as Prostaglandin H2 D-isomerase (PTGDS) and Cystatin-C (CSTC3) that account for approximately 40% of total CSF content after depletion vs 20% in non-depleted CSF. On the other hand, low and medium abundance proteins account for 59%, 54% and 36% in IgY14, IgYHSA and non depleted samples respectively, thus demonstrating significant enrichment of low- and medium-abundance proteins.

### Peptide fractionation

Peptide fractionation techniques are expected to increase the depth of analysis while possibly deteriorating experimental reproducibility. We set out to evaluate: (1) the gain in proteome coverage attained after peptide fractionation using offline reversed-phase; (2) the overall improvement of sample dynamic range; (3) experimental reproducibility in terms of peptide and protein identification.

**Figure 6:**
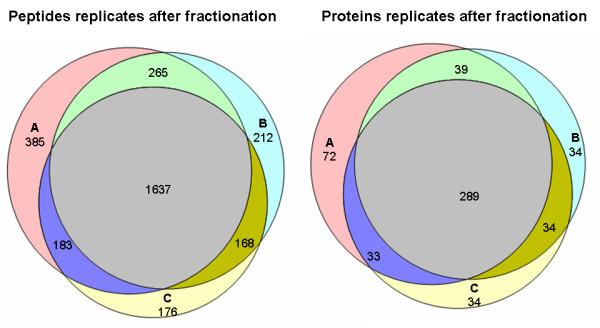
**Venn diagrams showing distribution of peptides (left) or proteins (right) identified in triplicate experiments after fractionation**.

Albumin-depleted CSF sample was fractionated into 30 fractions using preparative reversed-phase chromatography under basic pH. The numbers of confident peptide and protein identifications obtained from fractionated samples are summarized in Table 4. A total of 3,026 unique peptides were identified among the 3 replicates (1637 were common to the 3 replicates; Figure 6), corresponding to 535 non-redundant proteins (289 were common to the 3 replicates). Moreover RSD (relative standard deviation) was not increased when compared to unfractionated samples.

We compared the protein list generated with Mascot search alone using a target-decoy strategy or Mascot search combined with PeptideProphet and ProteinProphet validation analyses. CSF immunodepletion with IgYHSA column and analysis with 2DLC-MS/MS of one of the replicates led to the identification of 913 proteins with Mascot alone (FDR < 0.001). In contrast, with Mascot-TPP (PeptideProphet and ProteinProphet) strategy, a total of 947 proteins were identified, 402 of which were identified with high confidence and the remaining 545 identifications were grouped into one of the 187 protein groups for which members could not be distinguished on the basis of the peptides observed. The other replicates followed a similar trend.

The increased depth of analysis achieved with fractionation was also evident in terms of number of LAP detected in the sample. The number of proteins below 2 orders of magnitude from the most abundant protein as determined by APEX was used as a parameter to evaluate sample dynamic range following peptide pre-fractionation. Immunodepletion alone improved the number of LAP from 5 to 18 (Figure 2), whereas immunodepletion coupled with reversed-phase pre-fractionation further improved it to 53 proteins (Figure 2D).

## Discussion

Here we demonstrate that the reduction of sample complexity prior to analysis improves proteome coverage and the resolution of LAP. The combination of immunodepletion of the HAP and peptide fractionation is particularly attractive for "mining" CSF proteome. The objective of the study was to compare two immunodepletion methods with a simple and efficient procedure rather than identifying the largest number of proteins.

Protein inference following shotgun LC-MS/MS experiments is particularly complicated in biofluids, such as blood plasma or CSF, because of the frequent occurrence of protein families, multiple protein isoforms, and homologous proteins. The presence of peptides common to multiple proteins may lead to erroneous results at the qualitative and quantitative levels [18]. In the present study, we used ProteinProphet software with Occam's razor rules to reduce the protein list to the minimal set that can explain the peptides observed. To illustrate the effects of this strategy on our dataset, we compared the protein list generated with the Mascot search alone using a target-decoy strategy or Mascot search combined with PeptideProphet and ProteinProphet validation analyses. It should be noted that more than 86% proteins were identified with more than one peptide and that all peptide-spectrum matches (PSM) passed the > 0.95 PeptideProphet score. The enhancement of protein identification observed following CSF immunodepletion is in accordance with previous reports [11-14]. It should be noted that albumin depletion significantly improved protein identification in the present study. Moreover, 25 additional proteins were identified following 14-proteins vs. albumin depletion, while a previous study did not report increased identification with depletion of 6 proteins compared to albumin alone [13]. Another study compared two brands of 14 HAP depletion columns [19]. A large number of proteins were identified with both methods, but no quantitation was performed in the flow-through. Furthermore, in serum, improved protein identification appears to be related, but to a certain extent only, to the number of proteins depleted [20].

One of the most remarkable aspects of this study was the use of a spectral counting approach, namely APEX, to calculate protein abundance in the sample. Of note, the global dynamic range calculated with APEX was similar in the immunodepleted and the non-depleted samples. This finding was expected since the experimental dynamic range observed is a function of the MS dynamic range. It is in accordance with previous reports [13,14]. Nevertheless, we observed a significant improvement not only in the overall number of proteins and peptides identified, but also in the number of proteins with at least two orders of magnitude below the abundance of the most concentrated protein in the sample. These improvements were observed regardless of the immunodepletion system used, as only 4 LAP were additionally identified following 14-proteins depletion vs. albumin only. These results suggest that the ideal workflow should be elaborated individually for each study, taking into account number of identified proteins, as well as loss of non-target proteins. Dynamic range may possibly extend to 3 logs below that of HAP, if depletion methods were specifically designed to CSF and contained specific HAP like Prostaglandin-D-isomerase or Cystatin-C. Combinatorial peptide ligand library technology is another technique that was recently used to decrease dynamical range and thus increase LAP identification [7]. Several hundreds of new proteins were identified. However this method needs large sample volumes and extensive fractionation. When this method was adapted to small volumes, the total number of identified proteins was reduced to 530, which is quite similar to the number reported in the present study following fractionation (n = 520).

## Conclusion

Here we compared various methods attempting at enrichment of low-abundance proteins in CSF. This approach may be particularly useful in an effort to identify biomarkers for neurological diseases. The novelty of this study was to show the advantages and drawbacks of these methods side-to-side. We named and ranked proteins following two depletion strategies. Immunodepletion of high abundance proteins was shown to improve at least 3 folds detection of low abundance proteins, with good reproducibility. We compared dynamic range following immunodepletion alone or combined with peptide prefractionation. Offline fractionation using reversed-phase LC further increased 3 to 4 folds the overall number of proteins identified. According to the reliable identification and quantitation obtained with APEX algorithm, it may be considered as a cheap and quick alternative to study sample proteomic content, helping proteomics researchers to design more suitable analytical strategies. The optimal method should allow enhanced detection of LAP and prevent unspecific protein losses. These data also stress the urgent need for immunodepletion columns that specifically target the most abundant CSF proteins

## Materials and methods

### CSF samples

Using an atraumatic needle, CSF was obtained by lumbar puncture (2-4 ml per patient) from subjects attending the Department of Neurology at University Hospital Saint Etienne. CSF was collected in 12-mL polypropylene tubes (VWR), transferred on ice to the laboratory and centrifuged (3.000 × *g*, 10 min, +4°C). Fluid was aliquoted into 0.5 mL polypropylene cryotubes (VWR) and stored at -80°C. The study was approved by the local ethics committee of University of Saint Etienne. CSF samples from 5 ALS patients (Amyotrophic Lateral Sclerosis) aged 50-76 with clinically diagnosed or probable ALS following El Escorial diagnostic criteria were pooled and used for further analysis.

### Sample setup

The present study was devised using a single pooled CSF sample that was further divided into 12 aliquots. Each aliquot contained 780 μg total protein (Table 1). Nine of these aliquots were used for immunodepletion evaluation as follows: 3 were depleted of the 14 most abundant proteins (IgY14), 3 were depleted of albumin (IgYHSA), and the remaining 3 were not immunodepleted. The remaining 3 aliquots were depleted of albumin and further offline-fractionated using reversed-phase liquid chromatography under basic pH after protein digestion.

### Immunoaffinity depletion of highly abundant proteins

CSF immunodepletion of highly abundant proteins was performed using pre-packed liquid chromatography Seppro^® ^columns (GenWay Biotech Inc.). The term IgYHSA, refers to the column used for immunodepletion of albumin alone while IgY14 refers to that used for immunodepletion of albumin, IgG, α1-antitrypsin, IgA, IgM, transferrin, haptoglobin, α1-acid glycoprotein, α2-macroglobulin, fibrinogen, complement C3, and apolipoproteins A-I, A-II and B. Prior to injection on the column, each CSF sample was passed through a 0.45 μm pore size filter to remove particulates. As a result of the loading capacity of IgY14 columns, CSF aliquots subjected to these columns were further concentrated using a 3 kDa molecular weight cutoff (MWCO) filter (Millipore). A chromatographic column was set up on an ÄKTA Ettan system (GE Healthcare) and run following manufacturer's instructions. Finally, flow-through was desalted and proteins concentrated using a 3 kDa MWCO filter.

### Sample preparation for LC and LC-MS/MS

The final protein concentration of the depleted samples was determined by a bicinchoninic acid colorimetric assay (Pierce Biotechnology) using BSA as standard. Seventy five μg protein of each sample in dissolution buffer (0.1 M triethylammonium bicarbonate, 0.1%SDS) was reduced with 5 mM tris-(2-carboxy-ethyl)-phosphine for 60 min at 60°C. Free sulfhydryl groups of cysteine residues were then blocked with 15 mM iodoacetamide for 20 min at room temperature. Digestion with trypsin (Promega) was performed overnight at 37°C at a 1:50 enzyme-substrate ratio.

### Peptide pre-fractionation

To evaluate the impact of peptide fractionation following IgYHSA immunodepletion, trypsin-digested peptides were pre-fractionated offline by reversed phase liquid chromatography under basic pH conditions (RPb) on an ÄKTA system (GE Healthcare) using a 300 Extend C_18 _column (150 mm length × 2.1 mm ID, 5 μm particles, 300Å pore size; Agilent). CSF peptides were fractionated into 30 fractions. Peptide mixture dissolved in buffer A (25 mM NH_4_OH, pH9.5) was loaded onto the column and eluted with a gradient of 0 to 10% buffer B (25 mM NH_4_OH in acetonitrile pH9.5) over 3 min, then 10% to 28% buffer B for 8 min, and 28% to 45% buffer B for 4 min at 0.5 mL/min column flow rate. Fractions were collected at intervals of 30 seconds. Finally, acetonitrile was removed by evaporation and fractions were stored at -20°C until further use.

### Mass spectrometry

Prior to LC-MS/MS analysis, dried peptide samples were reconstituted with 0.1% aqueous formic acid. Peptide concentration estimates were extrapolated either from protein concentrations (non-fractionated samples) or from peptide absorbance at 215 nm during fractionation (RPb-fractionated samples). Approximately 200 ng of each sample was then loaded onto a 0.180 mm × 20 mm C_18 _precolumn Symmetry^® ^(Waters Corp., Milford, MA) coupled to an analytical C_18 _column (BEH130™ 75 μm × 10 cm, 1.7 μm, Waters Corp.) at 15 μl/min flow rate using nanoACQUITY Ultra Performance LC™ system (Waters Corp., Milford, MA). Peptides were separated in a 70 min gradient of 1-35% buffer B, followed by 15 min of 35-50% B (A = 0.1% formic acid in water, B = 0.1% formic acid in acetonitrile), at 250 nl/min flow rate. The column outlet was directly connected to an Advion Triversa Nanomate (Advion) fitted on an LTQ-FT Ultra mass spectrometer (Thermo). The mass spectrometer was operated in a data-dependent mode. Survey full-scan MS spectra (m/z 400-1800) were acquired in the FT with R = 100.000 at m/z 400 (after accumulation of a target value of 1e^6^). The five most intense ions were sequentially isolated for fragmentation and detection in the linear ion trap using collisionally induced dissociation at a target value of 50.000, 1 microscan averaging and a normalized collision energy of 35%. Target ions already selected for MS/MS were dynamically excluded for 30 s. Spray voltage and delivery pressure in the Nanomate source were set to 1.75 kV and 0.3 psi respectively. Capillary voltage and tube lens on the LTQ-FT were tuned to 35V and 109V. Minimal signal required to trigger MS to MS/MS switch was set to 100 and activation Q was 0.250. The spectrometer was working in positive polarity mode and singly charge state precursors were rejected for fragmentation. We performed at least one blank run before each analysis in order to ensure the absence of cross contamination from previous samples.

### Data analysis

MS raw data files were processed with Mascot Distiller (Version 2.3.2, Matrix Science, London). The resulting peak lists were searched with Mascot (Version 2.1) against the human International Protein Index (IPI) database (Version 3.71) concatenated with reversed IPI sequences. Search criteria were as follows: full tryptic specificity was required with up to 2 missed cleavage sites allowed; the precursor ion *m*/*z *tolerance was set at 20 ppm; the product ion *m*/*z *tolerance at 0.6 Da; carbamidomethylation (Cys) was set as fixed modification and oxidation (Met) as variable modification.

Peptide-spectrum matches (PSMs) were subjected to statistical validation with the PeptideProphet algorithm (TransProteomic Pipeline - TPP v4.3) using the accurate mass model option and the semi-supervised approach [21]. In brief, the expectation-maximization (EM) algorithm used by PeptideProphet to construct a Bayes classifier incorporates decoy peptide hits information from a target-decoy database search. All PSMs with PeptideProphet ≥ 0.95 were kept for further analyses. Finally, Occam's razor logic as implemented in ProteinProphet algorithm was applied to generate the most coherent list of proteins identified. Therefore, redundant protein entries were removed by clustering peptides by matching multiple members of a protein family to a single protein group and considering them as a single identification. Degenerate peptides were discarded before downstream quantitative analysis.

To gain insight into the protein profiling distinctiveness of the three protein depletion strategies, we used the modified spectral counting technique APEX (v1.2)[16]. This approach makes use of a machine-learning classification algorithm to predict peptide detectability. The program generates a correction factor for each protein (*O_i _*value), which is then used to predict the number of tryptic peptides expected to be detected for a given amount of a particular protein. Finally, spectral counts for each protein observed in a given run are corrected with their respective predicted *O_i _*value.The APEX abundance is therefore a modified spectral counting method in which the total observed spectral count for a given protein is normalized by expected (predicted) count (*Oi*) for one molecule or protein. In this regard, APEX abundance is considered the relative abundance of a particular protein with respect to all other proteins in the same sample.

Pattern similarity and quantitative analyses were performed using SuperHirn algorithm [22]. Briefly, SuperHirn performs peak detection and deisotoping followed by peak integration on each LC-MS run in order to build a peptide feature map. Multiple peptide feature maps are then aligned using 10 ppm precursor tolerance within a window of 60 second retention time.

## Competing interests

The authors declare that they have no competing interests.

## Authors' contributions

JB conceived and coordinated the study, acquired the data, and drafted the manuscript. AC designed the study, acquired the data, performed statistical analysis, and drafted the manuscript. CD acquired the data, performed the statistical analysis, and helped draft manuscript. NO acquired the data. EdO designed the study, and helped draft manuscript. JG coordinated the study and revised manuscript. MV designed the study, acquired the data, and helped draft manuscript. All authors read and approved final manuscript.

## Supplementary Material

Additional file 1 **Table S1: peptide identification after various depletion methods**. CSF samples were analyzed after depletion of 14 proteins (IgY14), albumin only (IgYHSA) or no depletion. LC-MS/MS analysis allowed identification of 1075 peptides validated with Peptide Prophet ≥ 0.95. Table shows list of peptides present (Y) or absent (N) after depletion. Click here for file

Additional file 2 **Table S2: protein identification after various depletion methods**. CSF samples were analyzed after depletion of 14 proteins (IgY14), albumin only (IgYHSA) or no depletion. LC-MS/MS analysis allowed identification of 189 proteins validated with Peptide Prophet ≥ 0.9. Table shows list of proteins present (Y) or absent (N) after depletion. Click here for file

Additional file 3 **Table S3: proteins abundance after 14 proteins depletion**. CSF samples were analyzed after depletion of 14 proteins (IgY14). Table shows list of proteins, number of peptides used for identification and APEX abundance score. Click here for file

Additional file 4 **Table S4: proteins abundance after albumin depletion**. CSF samples were analyzed after depletion of albumin (IgYHSA). Table shows list of proteins, number of peptides used for identification and APEX abundance score. Click here for file

Additional file 5 **Table S5: proteins abundance without depletion**. Non depleted CSF samples were analyzed by LC-MS/MS. Table shows list of proteins, number of peptides used for identification and APEX abundance score. Click here for file
